# Understanding the role of community resilience in addressing the Ebola virus disease epidemic in Liberia: a qualitative study (community resilience in Liberia)

**DOI:** 10.1080/16549716.2019.1662682

**Published:** 2019-09-11

**Authors:** O. Alonge, S. Sonkarlay, W. Gwaikolo, C. Fahim, J. L. Cooper, D. H. Peters

**Affiliations:** a Bloomberg School of Public Health, Department of International Health, Johns Hopkins University, Baltimore, MD, USA; b Liberia Center for Outcome Research on Mental Health, Monrovia, Liberia

**Keywords:** Community resilience, social capital, collective efficacy, Ebola, Liberia, low- and middle-income countries, shocks, health system strengthening

## Abstract

**Background**: There is an increasing recognition that community resilience plays a significant role in addressing health shocks like the Ebola virus disease (EVD) epidemic. However, the factors that constitute community resilience, and how these operate dynamically with other health system factors are less understood.

**Objective**: This paper seeks to understand key factors that constitute community resilience and their role in responding to the EVD outbreak in Liberia.

**Methods**: Key informant interviews were conducted between November 2017 and April 2018 with community representatives in Bomi, Margibi and Montserrado counties, and other national stakeholders involved in the EVD response in Liberia from 2014 to 2016. A national stakeholder meeting was conducted to verify and interpret information emerging from the interviews.

**Results**: Factors that were critical for addressing the EVD epidemic in Liberia were identified as: strong leadership, tight bonds and sense of kinship at the community level; trusted communication channels; and trust among various health system stakeholders. These factors facilitated collective actions within communities and helped to direct response initiatives from other levels of the health system to the community. Foreign assistance was seen as crucial for recovery and revitalization of affected communities. However, such aid is often not targeted at addressing critical challenges in a sustainable way, especially when the assistance is highly restricted to specific activities, and those activities are determined without consultation with local actors and community groups.

**Conclusion**: Efforts to systematically build responsible leadership and social capital at community level, including those that strengthen bonds in communities and trust across key actors in the health system, are needed to address health shocks like EVD outbreaks. Without building such capabilities in community resilience, it will be difficult to reap the expected gains from investments focusing on building physical capital and technical capabilities in health services and emergency preparedness.

## Background

The Ebola virus disease (EVD) epidemic of 2014–2016 was one of the most important public health threats this century. It was a crisis that challenged local governments and communities in Liberia, Sierra Leone, and Guinea, as well as governments around the world. The epidemic spread to affect over 28,000 individuals and led to over 4,800 deaths in Liberia [1,2]. Liberia’s health system was still recovering from nearly fourteen years of civil war and had limited capacity to respond when the outbreak hit. This was partly due to severe shortages of health workers, health facilities, pharmaceuticals, and other necessary materials and public health systems. The response was further hampered by poor roads, inadequate infrastructure, unreliable power and communications networks, and limited access to safe water supply [3].

A systematic analysis of interventions and their relationship to epidemic dynamics in Liberia showed that much of the decline in the epidemic curve came because of critical behavior changes within local communities and local public health measures, rather than depending on the bulk of international efforts that came after the epidemic had turned [4]. An important lesson was that strengthening health systems in Liberia required enhanced physical infrastructure, surveillance systems, and evidence-informed health services, and that the health care system (prevention and delivery) needs active community engagement strategies to foster community trust and collaboration [4].

Several authors have noted that listening to communities and supporting community-based interventions were increasingly important components of the EVD epidemic response, even as the international community focused on providing the infrastructure for clinical care and surveillance [4–6]. However, in the post-Ebola era much of the attention has reverted to strengthening government services, while neglecting the critical role that community-led activities played in turning around the EVD epidemic in Liberia. There is a need to understand the factors and characteristics within communities, i.e. community resilience, and how they operated dynamically with other health systems factors, to facilitate control and recovery from the EVD epidemic in Liberia. Such an understanding is necessary to maintain the gains, and potential of community resilience for strengthening health systems and responding to other health shocks in future.

The incidence of health shocks like EVD outbreaks and other large-scale catastrophic events (like war) may likely increase in the future, and the relevance of community resilience for addressing these different types of shocks need to be better understood. The recent 2018 EVD outbreak in an active war zone in the Democratic Republic of the Congo (DRC) has yet again highlighted the limitation of facility-based services and traditional public health tools alone in addressing health shocks. Despite the deployment of experimental vaccines and epidemiological tools, the incidence of EVD in the DRC doubled between September and December 2018, and the current outbreak is now the second-largest EVD epidemic on record globally [7]. Disease epidemics, and other types of shocks, occur within complexities of human interactions [8], and a disequilibrium with the immediate environment. It is not a coincidence that the EVD epidemic in the DRC emerges in the context of war and physical insecurity. Similarly, the origins of the EVD epidemic in Liberia have been attributed to increased population mobility across porous borders in search for work or opportunity in the context of economic insecurity and poverty [9]. Therefore, lasting solutions for addressing any shock need to consider the common factors within communities that make them vulnerable to multiple shocks such as war and disease outbreak at the same time. This is akin to understanding and treating the etiology of diseases, rather than just the symptoms in clinical medicine. To the extent that shocks have an underlying social basis, complementary tools that harness community resilience are needed for health systems and epidemiological interventions to work in addressing health shocks like EVD epidemics.

Resilience has been largely described in the health literature as a set of attributes or dynamic social processes that indicate the adaptive (ability to continue with ‘normal’ function, that is, an individual, organization or system continues to meet its objectives), absorptive (magnitude of stress that can be withstood while maintaining ‘normal’ function) and/or restorative capacities (ability to get back to ‘normal’ function) in response to a health shock [10–17]. Other authors have emphasized the significance of defining positive health trajectories as outcomes of resilience, and the human agency to engage and change both the internal and external contexts that give rise to health shocks or the maintenance of adverse outcomes following the health shocks (transformative capacity) [10,18,19]. This paper describes the experience in Liberia of identifying and building resilience at the community level in the context of the EVD epidemic, and against the background of a history of protracted civil war in that country. A community is defined as a ‘group of people with diverse characteristics who are linked by social ties, share common perspectives, and engage in joint action in geographical locations’ [20], with emphasis on the geographical location given how EVD spread within a contiguous space linked by person, place and time. The paper seeks to understand key factors (set of attributes) that may constitute community resilience and their role (adaptative, absorptive, restorative and transformative capacities) in responding to the EVD outbreak. It explores promising strategies for strengthening these factors in responding to health shocks more broadly. It is hoped that this paper contributes to discussions about strategies that strengthen community resilience and link communities to public health systems in preparation and response to future shocks. In particular, it is hoped that critical gaps in poor and vulnerable communities in low- and middle-income countries (LMICs) can be addressed by the lessons learnt from this paper.

## Methods

### Study design

A qualitative study design was used to explore factors, characteristics and responses within communities that facilitated recovery from the EVD epidemic in Liberia. How these factors interacted dynamically with other health system factors was also explored.

### Study settings

The study included two major hubs involved during the EVD epidemic in Liberia: communities in border areas with neighboring West African countries, and slums settlements in large urban settings. The study included participants from communities in Bomi, Margibi and Montserrado counties of Liberia, which were more affected counties during the EVD outbreak. Bomi county lies on the border with Sierra Leone and includes Tubmanburg that had high rates of EVD transmission. Margibi county includes Dolo’s Town which was a major epicenter for the transmission of EVD. Montserrado county includes informal settlements and slums in the city of Monrovia such as West Point, New Kru Town, Soniwen, Doe Community and Gibraltar communities.

### Participants

Participants were purposively selected to represent community leaders and members from pre-identified communities in Bomi, Margibi and Montserrado counties, and other stakeholder groups involved in the EVD response in Liberia from 2014 to 2016. These other stakeholder groups include frontline health workers, representatives from Bomi, Margibi and Montserrado County Health Teams, Ebola Survivors Network, relevant non-governmental organizations (NGOs), and key departments within the Ministry of Health (MOH), the National Public Health Institute of Liberia (NPHIL), the Ministry of Gender, Children and Social Protection, and professional associations including those for nurses, physician assistants and pharmacists. The stakeholders were selected primarily to include organizations and groups that played a key role during the EVD epidemic, and specific individuals within those organizations or groups that were recognized for their leading roles.

### Data collection methods

The data collection approach included key informant interviews (KII) and a national stakeholders meeting. A structured interview guide consisting of open-ended questions was used to facilitate the KII. The interview guide focused on five key topics (Box 1).

**Box 1.** Themes covered in the interview guide.

1. How do different type of shocks affect communities?
2. How did the communities respond to the EVD epidemic, and how was the response compared to the civil war?
3. What are the community resources and factors that can limit shocks, and how were these made available and used (or unavailable and unused) for addressing EVD?
4. What are enablers and inhibitors of these community resources and factors?
5. What are some potential strategies for strengthening these community resources, characteristics and factors? What are some of the gaps in our understanding of the role of these community factors in addressing shocks like EVD?

Four research assistants were recruited to conduct face-to-face interviews, and were trained for two days in research ethics, interview techniques and practice of the interview guide. All four research assistants were university graduates and had experience in conducting qualitative interviews in Liberia. The interviews were conducted between November 2017 and April 2018 and continued until data saturation was reached. All interviews were conducted in English. Following each participant’s consent, the interviews were digitally recorded, and handwritten field notes were taken by the trained interviewers. The digital interviews were later transcribed and merged with the handwritten field notes. To ensure accuracy in written transcripts, two of the study investigators listened to the audio recordings, reviewed and cleaned the data where appropriate.

A national stakeholders meeting was conducted in February 2018 with the stakeholders included in the KII. The stakeholder meeting was used to corroborate and interpret the information that was emerging from the KIIs, and to provide recommendations on strategies for strengthening community resilience in the Liberian context.

### Data analysis

The data was coded using Nvivo software, and the analysis was guided by a coding grid developed by the study investigators. The data was first coded based on types of shocks (EVD and war) to draw parallel in community response to EVD compared to the civil war. There are a wide range of meanings of community resilience [10,14,21,22], as well as related concepts of health systems resilience [23]. Rather than focus on a specific definition of community resilience in this analysis, Patel and colleagues’ [14] model of community resilience was first applied to unpack the dimensions of community resilience relevant within the Liberian context. This model includes nine broad elements of community resilience (local knowledge, community networks and relationships, communication, health and health services, governance and leadership, resources, economic investment, preparedness, and mental outlook) which were terms employed in the first round of coding. Based on the emergent themes from the data, a second round of coding was conducted to capture the dynamic interactions among the terms used in the first round of coding while examining how communities responded to the EVD outbreak. Where relevant, quotes were double coded to identify linked themes, and the data was triangulated via team discussions and comparisons with notes and reports from the stakeholder meeting.

## Results

Data saturation was reached at a sample size of 36 interviews (Table 1). Participants included community members, local chiefs, survivors, representatives of government ministries and NGOs, academic, religious leaders and policy-makers. All the 36 interviews were conducted in person. Mean time per interview was 35 minutes (range 30 minutes – 1 hour).

**Table 1.** Participants included in key informant interviews.

Organization/groups	Number of people interviewed
Community Residents	9
Ministry of Health	9
National Public Health Institute of Liberia (NPHIL)	3
County Health Team/Frontline Health Workers	4
Professional Groups (Physician Assistants, Nursing and Pharmaceutical Associations)	4
Ebola Survivor Network and other local NGOs	3
Ministry of Gender, Children and Social Protection	1
International Organizations	2
Academic Institution	1
**Total**	**36**

## Parallels in community responses to shocks: civil war and EVD epidemic

Responses identified as common to both the war and EVD outbreak in Liberia could be mapped under five themes, including: distrust, fear, death, psychological trauma, and community cohesion (Table 2).

**Table 2.** Some parallels in how communities responded to both EVD and civil war shocks.

Themes	Ebola	War
Distrust	of health system, of information channels	of government, of political institutions
Fear	of direct transmission	of attack, loss of life
Death	EVD as an ‘unseen enemy’ that resulted in loss of life	war as a ‘known enemy’ that resulted in loss of life
Psychological trauma	due to loss of loved ones, fear of transmission, lack of closure due to burial practice	due to flashbacks of war, brutality, post-traumatic stress disorder
Community cohesion	once people understood the threat of EVD, they began to unite as a community	community member cared for each other in the wake of the war

A higher number of deaths and increased level of fear were prevalent during both shocks, relative to the period before the shocks (Table 2). During the EVD outbreak, communities feared direct transmission of EVD, which was perceived as an ‘unseen enemy’. In contrast, communities feared attack by ‘clear and known’ enemies or sometimes enemies that may be disguised as community members during the war. Both of these shocks led to the depletion of economic resources and infrastructure, limited access to social networks, and resulted in significant psychological trauma among survivors; and for many, unresolved trauma related to the civil war may have been further exacerbated during the EVD outbreak as reported by one participant,

“The lingering effect of the civil war … we still had issues as regard to community understanding, coping mechanisms in communities … .So, I think the Ebola outbreak further disrupted that [the] development of communities that had linger over a long period of time.” (MOH official)

There were other responses that manifested differently during both shocks. For instance, distrust by community members during the civil war was directed at the government and political institutions depending on warring factions in contrast to EVD, where distrust was directed towards specific health system actors e.g. the Ministry of Health (MOH) which further contributed to the misinformation regarding EVD risks. As some participants responded,

“Since they did not have the truthful information about what really Ebola was, how it occurred or how can it be addressed or prevented, it created a lot of issues and one of those issues was lack of trust. Because if you did not know exactly what it is, then you do not trust anyone anymore.” (Community member)

“There were several places … where people went, and they drove them from the communities and I am telling you that they beat some of our staff up because they did not know or did not trust us anymore to give them any information. So, that lack of trust created a lot of problems for us.” (MOH official)

Participants reported a sense of strong community cohesion during both times of shock. Once the issue of misinformation was addressed with the EVD outbreak, and most communities understood the threat and nature of the disease, they bounded together to overcome the stigma associated with EVD transmission and respond to the outbreak. Similarly, most communities supported one another to recover from the psychological, physical and economic impacts of the war by sharing economic resources and other kind of resources.

Finally, while outcomes, including distrust, fear, death, psychological trauma, and community cohesion were common in both rural and urban areas in response to both the war and EVD, participants from urban slums reported that these outcomes were more widespread following the EVD outbreak than during the war. They reported that the war in the urban slums was targeted to specific ethnic groups, unlike the widespread impact of EVD on multiple groups, including individuals of different ethnicity, religious, and social backgrounds that reside in the same geographical areas.

## Unique responses to EVD shock

Compared to the war, several themes were unique to the impact that EVD had on communities, and their responses to this impact. First, EVD was associated with a widespread stigma that was not observed during the war. For many, the stigma stemmed from rumors and misinformation regarding how the disease was transmitted, and who was susceptible to contracting the disease. The misinformation and stigma were not limited to a specific demographic group; for instance, it equally affected both educated and non-educated individuals. As one participant described,

“One of the gaps that I see, and people still talk about is that people still believe that Ebola is not a natural disease but a manmade disease … The communities still need more awareness, more anti-stigma messages, more education on EVD.” (Frontline health worker)

“If you don’t address the rumors … you don’t expect the community to be actively involved because they are blind to the whole thing … They don’t know what to do … Especially the chiefs, they didn’t know what to do because they didn’t understand. So, that how actually it impeded the response efforts.” (MOH official)

Second, EVD had a significant impact on cultural norms and expectations, compared with the war. Communities were instructed to change their burial practices to avoid touching deceased bodies because EVD can be transmitted via contact with the dead. For many, these revised burial practices led to psychological trauma, and a lack of ‘closure’ regarding the loss of a loved one. The need to carry out rituals for healing, and in preserving one’s place in the afterlife stood in sharp contrast to steps needed to stem the epidemic. As some participants described,

“Every ethnic group or community have their own practice … some groups will bathe the dead and the bath water will rub it on themselves and they do consider it as blessing.” (NGO official)

The EVD also impacted other social practices in the communities. For example, touching and handshaking are common among Liberian communities, yet individuals refrained from such touching to avoid transmission during the EVD outbreak. The outbreak led to changed norms in how communities washed and prepared their food, and prevented families from eating collectively. Moreover, many participants expressed a fear of hosting and caring for family, friends and neighbors affected by EVD, as one participant described,

“Because when our brothers and sisters were sick, they told us not to touch them. If we were giving them food, it was by dragging a stick with the pan or bowl. So, it made us to feel bad in the community. It was too bad.” (Local chief)

Finally, participants reported that they felt they had agency to mitigate the risk of EVD which they did not have during the civil war. They reported feeling a sense of control as a community on ways to reduce EVD risks, and employing preventative measures to decrease the spread and impact of the disease throughout their community; unlike during the war, when participants reported feeling a sense of helplessness towards both the direct (e.g. brutal attacks) and indirect (e.g. economic loss) impacts of the war.

## Community resilience: barriers and facilitators to community responses to EVD

“I think in disaster preparedness it is always said every disaster begins in the community and ends in the community.” (MOH official)

Key factors that constituted barriers and facilitators to communities in adequately addressing the spread of EVD were identified, and the dynamic changes in these factors over the course of the EVD outbreak was used to explore the role of community resilience in addressing the epidemic in Liberia. These factors include:

### Leadership, trust and communication

Misinformation regarding Ebola risk and transmission was prevalent at the onset of the outbreak. This misinformation affected most communities in putting measures in place to stop the spread of the virus and working effectively with other stakeholders within the health system. As described by one participant,

“Misinformation from so called community’s ‘experts’ who thought they knew the answer [was a gap in the response]. Wild[ly] spread rumors and distrust between the health care workers and the response team … those were issues that held the community back and made them more vulnerable to these shocks and threats.” (NPHIL official)

In communities with strong and trusted local leadership, and close networks among community members, the response to the outbreak was effective when the right information was provided through these trusted leaders, because they were viewed as trusted sources of information. The reverse experience was the case in communities with perceived weak leadership and connections among community members, as described by one of the participants,

“Well some of community gaps that were experienced were the leadership were (sic was) not effective. For some communities the bonds were not created so communication was lacking. You could not get the facts on time when it was needed and also based on past experiences within the communities, people were not talking to each other.” (Ebola survivor)

The local leadership represents a major source of information and knowledge, and controls trusted communication channels within the communities. Community’s trust in local leadership was a crucial factor in addressing the EVD outbreak, especially where these leadership structures could access the right information about the disease. It really did not matter if these leaders or elders were educated or not; the key issue was whether these were trusted leaders, effective communicators and had access to the correct information about the disease. As some participants described:

“If you think communities [leaders] are illiterate and based on illiteracy you think they are ignorant, then you are causing yourself a major problem. Because illiteracy does not equate to ignorance. I think what it is, is trust. And if people trust you they will do whatever you tell them to do.” (MOH official)

“Because some places like a chief who is almost like a clerk who does almost all of the writings for people went there and told the community members that the people [health workers] were lying, Ebola was not real and in fact they should not allow health workers to enter the community because they were the ones taking [introducing] Ebola within the community … and then people saw him to be the most educated guy in the community it became a problem.” (Member of a professional association)

The emphasis on trust between community members and leaders, and having trusted community leaders disseminating accurate information on Ebola risk and transmission in mitigating the spread of EVD was consistent across stakeholders interviewed, including community residents, MOH officials and members of professional association. The distinction between illiteracy of community leaders, which did not have any significant impact on the spread of EVD, and ignorance or failure to acknowledge accurate information about the disease, which had a major impact on the spread of EVD was mainly noted by stakeholders from the MOH.

The mistrust between government and communities was further exacerbated by weak community leadership in some of the worst affected communities. The belief that EVD was a manmade disease or a rumor perpetuated by the government prevented effective communication and messaging. In response, campaigns and messages to reduce misinformation were developed by the MOH, Ministry of Information and other local and international organizations. These campaigns and messages were then disseminated to chiefs and leaders, who further communicated the messages to their community members. These campaigns were successful in communities with trusted leaders, and the process of messaging further fostered trust and led community members to change their daily practices (e.g. handshaking, food preparation) to adhere to transmission prevention. As explained by some participants,

“Initially people had different room [rumors], myths and beliefs about what promoted Ebola. Ranging from witchcraft all the way to mosquitoes. And then it got to a point where information was provided [by MOH] that counteracted some of that. And the more accurate information was shared with the communities and then there was a question about making sure that the information was being shared by the right messenger. And then after that the communities moved into a stage of acceptance and then moved on to the stage of overcoming Ebola.” (NGO official)

“I think one of the things we had to overcome was the distrust that existed between the communities and the government. So, for a long time it was very difficult for us to get penetration [go through] with the health communication messages that we were trying to get because it was being delivered by the government … it took a lot of work from NGOs and local advocacy groups, community groups and others, local champions to get that work done.” (NGO official)

In communities with strong leadership, and where those local leaders were actively engaged, the leaders fostered partnerships with NGOs and helped to build trust and facilitate the entry of government and health organizations into the communities, as described by some participants,

“The people [local leaders] understand people in their communities more than you and myself. So, that’s their strength. That’s one strength that they have. All you need to do is put the mechanism in place in the way that you will utilize the skills that are in them.” (MOH official)

“We engaged them [local leaders], got the chiefs on board … we met with them. The chiefs, the elders, religious leaders we met with them and volunteers. We even went from district to carry the message … as the result … They got involved. They took ownership. They helped with the response. So, that’s how he community got organized.” (MOH official)

### Community bonds, economic resources and collective action

Strong bonds and kinship among community members facilitated community ownership of the problem and collective actions to address the spread of the disease. Several participants highlighted the importance of community ownership of the problem and employing a bottom up (rather than top down) approach to the response. For example, many communities with a strong sense of kinship began their own surveillance and response initiatives prior to any intervention from government and international organizations or establishment of Ebola Treatment Units (ETUs). These community activities were found to be a timely, and an effective approach to transmission prevention as compared to waiting for outside health agencies to intervene even though it relied heavily on meagre economic resources available within the communities, as demonstrated by the following comments from participants:

“They work together. Initially there were [was] distrust, but they began to realize very quickly that they needed each other to survive against this treat [threat]. And we saw communion [community] bonding right after Ebola.” (NPHIL official)

“Once community stepped in and started keeping an eye on community members when they got sick and making sure that they were not moving from place to place until the right authorities took custody of them. So bottom line communities play a major role, had it not been for communities it would have been impossible to fight the virus.” (County health team member)

“The opportunity is that the community is capable of solving problems cost effectively if you allow them [community] to own the problem. If you allow them to propose the solution and you work along with them to support them we will be able to solve many other problems in the country. And it will cause [cost] us far less because the communities see themselves as part of the solution.” (NPHIL official)

“The international community did not come to the aid of Liberians until the epidemic was trending downwards.” (County health team member)

All participants, including MOH officials and community residents, cited community bonds as one of the most valuable resources for addressing the EVD outbreak because this bond facilitated collective actions such as pulling together economic resources to take care of the sick and getting them to the health facilities.

“This is the most important resources, the people. Those are the resources that can be expected to attack any shock.” (NPHIL official)

“I think the best to have is the people themselves. The best resources they have is how well they can talk to each other and how well they can relate to their neighbors and so forth.” (MOH official)

However, it was only the community residents and non-MOH participants that drew attention to the role that lack of infrastructure and economic resources played in hampering the collective actions that emerged as a result of the community bonds. As some of the participants described:

“For instance, here we don’t have any roads. When someone gets sick we gather ourselves in the community and put the person in a hammock and take them to the nearest clinic. This can involve hours of walking to the facility.” (Local chief)

The need to build infrastructure such as roads during periods of stability was emphasized, and the role of such infrastructure in building resilient communities was described. As one participant explained:

“Another factor would be from the bigger level is the political will of those who are making decisions and when it comes to matters affecting the people, they must have the will to improve the facilities, they must have the will power to educate the people on the line with that you can have a healthy and strong community.” (Member, professional association)

Moreover, some communities were still recovering from economic declines from the impact of the civil war, particularly on the mining and agricultural sectors. A strong theme to emerge in the interviews was the connection between the need to restore these industries for economic resources to support future collective actions and to strengthen community resilience:

“Right now, out of the community, the support we need as Ebola is gone, is agricultural support because we are farmers … We need support from the government through the Ministry of Agriculture as local farmers. Because after the crisis there has been food shortage. Everyone must go Kakata to buy half bag of rice but not everyone can afford it. But if government can bring agricultural materials with a policy of compulsory farming, the issue of hunger will reduce in this country.” (Local chief)

### Health services delivery system

During the EVD outbreak, the health services delivery system was severely strained. Many health facilities were overwhelmed, had limited space, health personnel and capacity to attend to both emergencies and routine services at the same time. Many health facilities closed for long periods during the outbreak’s initial stages. Others were shut down because they became sources of infection. Fear of EVD transmission caused many health professionals to stop going to work, which further overwhelmed those who were responding. As noted by one participant,

“A lot of the health facilities were closed [shut down] at the time. Clinics in those neighborhoods were afraid to take patients in … Because people were afraid, health workers were afraid.” (NPHIL official)

The strain on the health services system during the epidemic reflect weaknesses in the system that predate the EVD outbreak, especially at the community levels. There were pre-existing issues such as lack of access by community members to functional health facilities, shortages of human resources for health, and inadequate supplies of other health services inputs before the outbreak. The outbreak only further worsened these issues. As some of the participants described,

Participant: Right now, we don’t have anything. When we get money, we buy chloride, soap and we wash our hands. And that is not medicine for us to take in [and there is no medicine for us to dispense].

Interviewer: So, you people don’t have anything at all?

Participant: We don’t have anything.

Interviewer: What are some of the resources that are available but not many and are unused by the community?

Participant: We don’t have anything unused.

(Local chief)

“Also, the health facilities have a lot of gaps because they are not in every community, people have to walk to the communities before they can get any information if they do want to hear.” (Member, professional association)

Several emergency programs were put in place to treat persons with EVD and to prevent further transmission. For instance, the Ministry of Health developed the Infection Prevention and Control Program, which facilitated quarantines, triaging, and risk communication, including provision of hand pumps, latrines, and sanitation protocols as well as hand wash stations and buckets to communities. Mental health support workers, while not available to all affected individuals, travelled to communities to support victims and survivors. As one participant described,

“The social workers, mental health clinicians went into the different communities, especially communities that were highly hit, they went into those communities interacted with the communities’ dwellers and launched what we called the community healing dialogue as a way of de-traumatizing those community members who were really affected by the Ebola crisis and tried to bring about stability. (Ebola survivor)

Several participants emphasized the need to have these health services programs available during periods of stability and recognized that they are critical for the communities to respond adequately to future health shocks. Respondents also noted the need for communities to continue to learn and adapt interventions that were beneficial during the outbreak, and not revert to old harmful practices, as some described:

“I wish we could have maintained some of the good practices like that of washing hands and making sure that we adhered to some of the protocols but now as it stands people are already falling back to their old practices, it is so unfortunate.”

“It took months to get people to accept the dead body management team to come in and bury their dead and take precaution(s) instead of them carrying on their normal practices. I don’t know by this time whether people have gone back? There’s a possibility that people have gone back to these practices.”

Some participants reflected on the distortions to the health system caused by a large influx of restricted funds from foreign donors during the EVD outbreak, which goes mostly to address emergency needs. For instance, millions of dollars were used to build temporary ETUs, which were subsequently torn down. Participants further emphasized the need for flexibility to be given to MOH and recipient communities in how these emergency funds could be allocated to strengthen the health services delivery system, especially at the sub-national level. As seen in the following quotes,

“I think external support should not be targeted to specific things when you are going through crisis. Whatever it is, it could be funding, it could be material …, there should be a flexibility clause … [for EVD] the funding was so restrictive, you couldn’t do so many things … We spend millions of dollars building temporary ETUs. Even though we knew that we had broken the chain [i.e. stopped the epidemic], that we wouldn’t have used these ETUs …, still we had to spend the money because the funders insisted this is what they wanted to do. So, at the end of the day it made no impact on the system, you know. So, there was a waste, so much wastage in the process …, external support should target what is needed in a community, or in that country in order to help build the system and not to cause a major, major crisis in the system.” (MOH official)

Finally, while the analyses of the facilitators and barriers of community resilience presented so far applied equally to both rural and urban areas, a unique distinction lies in the significant role that community health volunteers played in surveillance, messaging and quarantine activities in urban slums of Monrovia such as West Point. These community health volunteers comprising of youth leaders had surveillance, health messaging and quanrantine responsibilities in designated health zones within the slums in Monrovia and they had direct communication lines to the Infection Prevention and Control Unit within the central MOH in Monrovia, and access to the health facilities in their catchment areas. The co-location of the activities of these youths and the central MOH in Monrovia further facilitated a rapid exchange of health information and resources between the MOH and the urban communities. These youths later formed the core of the health facility development committees that served as a critical linkage between the health facilities and the communities after the EVD epidemic was brought under control.

## Discussion

Liberia’s recent history of war and EVD outbreak created an opportunity to draw parallels in how communities respond to different types of large-scale catastrophic events or shocks. Whereas factors such as distrust, fear, death and psychological trauma were common responses and consequences of both shocks, individuals and communities perceive a stronger agency to control factors related to the spread of the EVD outbreak and its consequences, compared to the war. This human agency has been described as transformative capacity in defining community resilience [18,22,24–26]. Other definitions of community resilience include its description as adaptive, absorptive and/or restorative capacities to health shocks [10–17], as described earlier. Both the war and EVD undermined the adaptive and restorative capacities of communities in Liberia by depleting economic resources, infrastructure, and access to social networks; although they both fostered social solidarity which would have facilitated the communities’ capacity to absorb the shock of the EVD outbreak following the war. The emergence of the EVD outbreak following the war in Liberia highlights how the history and aftermath of war increases the vulnerability of communities to other future health shocks, limits their adaptive and restorative capacities to respond and rebound quickly from these shocks, while strengthening their ability to withstand or absorb the shocks at the same time. This finding highlights the complex and multidirectional interactions between the history of war and other health shocks like EVD as they influence community resilience. It further highlights the need for multiple approaches in strengthening community resilience, including approaches that seek to provide economic resources and infrastructure while also facilitating community healing and the cognitive abilities among community members to learn and self-organize following a shock.

The stronger sense of agency with EVD outbreak compared to the war further suggests that first, community resilience may be measurable, since individuals and communities can compare their levels of resilience in response to two different types of shock, and such measurement may facilitate effective design and implementation of strategies to strengthen community resilience. Second, community resilience may vary by the types of shock. For example, while communities may be resilient to everyday challenges for survival, they may be more vulnerable to large-scale catastrophic events like a severe disease pandemic or war, and within categories of shock e.g. large-scale catastrophic events, levels of community resilience may also vary. Thus, in understanding community resilience or how to strengthen it to address shocks, it is important to first clearly define and characterize the type of shock in question because strategies for strengthening community resilience may vary for different types of shock.

This study identified factors which may constitute domains of community resilience, consistent with existing literature [10,14], and provides new descriptions for some of these factors. These factors include a strong leadership, bonds and sense of kinship at the community level; trusted communication channels; and trust among various health system stakeholders. These factors, broadly classified as leadership and social capital hereafter, were reported to have played a more important role in mitigating the spread of EVD than funder-led short-term initiatives which were funneled through facility-based health services. While these findings are consistent with other studies [4–6], this study provide further insights and confirmations from community respondents and survivors on how these factors operated dynamically to facilitate recovery in Liberia. These factors operated mainly by facilitating collective actions within communities, and effective dissemination of response initiatives from other levels of the health system. Indeed, different stakeholders, including both MOH officials and community leaders, noted that future efforts to address shocks should target strengthening leadership and social capital at the community level, in addition to investment in infrastructure and health services delivery systems (physical capital) and provision of livelihood and economic opportunities. The complementarity of social capital, physical capital and opportunities for economic development has similarly been identified as critical for addressing social challenges in other contexts including the inner-cities of the USA [22,27]; thus, highlighting the need to prioritize efforts that address social capital in strengthening community resilience within the context of health systems strengthening and emergency preparedness. Respondents explained that communities with strong leadership and social capital before the EVD outbreak were able to recover more quickly, limit the spread of the disease and minimize the consequences of the diseases by initiating behavioral changes and making the most efficient use of limited resources available within the health system. Respondents further highlight that quick recovery did not occur in other communities lacking leadership and social capital despite the availability of similar health system resources.

Social capital has been defined as ‘the rules, norms, obligations, reciprocity and trust embedded in social relations, social structures and society’s institutional arrangements, which enable members to achieve their individual and community objectives’ [28]. Several authors have suggested that the existence of small groups, and formal bonds among these groups with established norms and trust (that is, bonding social capital), facilitate mutually beneficial cooperation among their members [27,29–31]. The products of such mutually beneficial cooperation, including trust and reciprocity, are self-reinforcing and cumulative, yielding exponential positive benefits including strengthening community resilience and economic development as time progresses [27,31]. As we found in this study, the existence of small groups with formal bonds e.g. those belonging to the same clan or religious affiliations facilitated effective community surveillance, contact tracing and continuous community mobilization that were crucial for limiting the spread of EVD, which further reinforced the sense of kinship within the members of these groups. This finding further highlights how community resilience may mediate the effectiveness of traditional public health tools in emergency preparedness and recovery from health shocks, and how social capital may be reinforced within communities. Several authors have suggested that social capital may be reinforced within neighborhood and communities by activities such as those that establish and facilitate productive engagements in civic and religious groups, cooperatives and farmers’ association in agrarian societies, and educational programs through schools and various community groups [32–36]. They however cautioned that these activities must be implemented in ways that they do not reinforce harmful power imbalances and social inequalities that may be embedded in the communities, or negatively impact individual liberties [27,30].

Similarly, activities that cultivate effective grassroot leadership e.g. formation of action-oriented community-based committees, support of recognized and responsible leadership councils, and establishment of trusted, local communication channels (e.g. through community-based news media or network of family or neighborhood leaders) could strengthen community resilience. For example, this study found that action-oriented committees in slums of Monrovia comprising youth leaders played significant role in facilitating effective coordination of recovery effort and disseminating accurate information on the EVD outbreak. Some of these action committees later morphed into branches of the district administration and are strengthening cooperation within the communities and with the central administration. Respondents further noted that communicating health promotion and disease prevention messages through recognized community leaders, creating partnerships between government and communities during times of peace to promote trust, providing information and resources that allow communities to take ownership of response efforts could help strengthen community resilience.

Foreign aid to support countries in responding to health shocks like EVD is crucial for recovery and revitalization of affected communities [37]. However, such aid may distort the health systems in situation where they are not adequately targeted to address critical challenges in a sustainable way as was suggested by some of the respondents in this study. For example, a range of stakeholders interviewed in this study commented that building temporary treatment units after they are not needed, and then tearing them down was counterproductive to strengthening health systems at the community level. Such mismatch between resources and activities is likely where the funding is highly restricted to specific activities, and where those activities are decided without consultation with local actors and community groups. Other studies have shown that health system functions prioritized by global and national actors improved significantly more compared to those prioritized by community leaders in the wake of the EVD epidemic in Liberia [4,38], even though local actions at the community level were the most significant for addressing the epidemic as suggested in this study. Whereas the need to mitigate corrupt practices and perverse incentives in administering foreign aid is understandable, this study however suggests that the lack of flexibility in decision-making and allocation of funds could limit recovery of communities from health shocks. Also, once funds are committed to specific activities, mechanisms that allow for continuous consultation with local actors during the implementation of those activities could have helped to re-focus and re-orient the resources. Based on the principal-agent theory, several studies have identified that giving latitude to local actors in allocating funds within the bounds of pre-specified targets could help in achieving goals such as improving coverage of health services and health inequities in low and middle-income countries [39]. Such principles should be extended to administering foreign aid during emergencies as well. Providing flexibility to local actors could allow resources to flow into supporting activities that reinforce social capital both during emergency and non-emergency periods, while also facilitating targeted and relevant development of the physical capital needed for providing sustainable health services in specific contexts. Other activities for strengthening efficiency in fund allocation and effectiveness of interventions, including the performance-based approach, could similarly be considered.

The finding that learning and adaptation is critical for strengthening community resilience is consistent with theories and frameworks for understanding community resilience in the global literature [40–42]. Public health institutions such as the Ministry of Health and the National Public Health Institute of Liberia (NPHIL) were identified as playing a crucial role in addressing the EVD outbreak in Liberia, and creating platforms for interorganizational structure and network (that is, networking social capital) [31] that further facilitates learning and adaptation. The NPHIL was established with strong donor support, at the height of the EVD outbreak, and it focuses on disease surveillance and control. Since after the EVD outbreak, the NPHIL has played leading role in the detection and control of other public health emergencies in Liberia, including outbreaks of Lassa Fever in recent times. There is a need for continual capacity building and support of public health institutions such as NPHIL that are positioned to address disease outbreaks at population levels to build more resilient health systems. As noted earlier, such efforts are complementary to activities for strengthening community resilience.

Comparing rural and urban settings, bonding social capital played a significant role in fostering community resilience in response to the EVD epidemic in both settings. However, networking social capital appeared to have had a more signficant impact in the urban setting given the prominent role of community volunteers in the urban slums, the co-location of their activities and the central MOH in Monrovia, and the legitimate and direct access they had to information and resources from the central MOH. This observation may explain why the EVD epidemic was more rapidly brought under control in the urban slums compared to some isolated rural areas, and highlights the synergistic role of both bonding and networking social capital in fostering community resilience [31,43]. Indeed, the community-led activities in the urban slums were later extended to other areas within the city of Monrovia and served as important lessons in organizing response to the EVD epidemic in urban areas for future public health crises [44].

The ability to diversify economic production between small-scale mining and farming has been previously identified as important for strengthening community resilience in the face of EVD in rural areas of Liberia and Sierra Leone [45,46]. Findings from this study suggest that rural areas of Liberia whose farming and mining activities were both affected by the civil war may have been less resilent to the EVD epidemic because of the direct impact of the epidemic on the population’s ability to engage in these livelihood activities. Groups formed around farming and mining activities are also essential for facilitating bonding social capital in rural areas, and it is probable that communities were such groups existed before the EVD epidemic may have been better prepared to coordinate a community response to the epidemic.

This study provides important lessons on the role of community resilience in preparation and response to shocks. The two-year time lag between the end of the EVD epidemic (in 2016) and when this study was conducted (2018) is a major limitation of this study, and the study findings are subject to recall bias. However, this time lag has also provided respondents time to reflect on what has happened, which may not be doable during the crisis itself. This study considered adequate and varied sample, and was conducted in both urban and rural settings so as to facilitate the transferability of the study findings to other similar contexts in LMICs. The study investigators were not involved in the EVD response activities in the selected communities to limit the impact of any preconceptions on the study findings.

## Conclusion

Community resilience, including leadership and social capital, is crucial for addressing health shocks like EVD, and facilitating recovery of communities from such shocks. However, efforts to proactively strengthen community resilience do not attract the same level of programmatic and financial support, as compared to investment in emergency and facility-based services. Community resilience addresses health shocks by facilitating collective actions within communities, and effectively targeting resources and response initiatives from other levels of the health system to the community. Where funder-led short-term initiatives dominate recovery efforts, the role of community resilience becomes more important particularly in contexts with weak infrastructure and capacity to coordinate response. Efforts to systematically build social capital and responsible leadership at the community level, including those that strengthen bond among groups in communities and trust among various actors, are needed to address health shocks like EVD in future.

## Paper context

The mechanisms by which community resilience contributed to addressing the Ebola epidemic in Liberia are less clear. This paper showed that factors like leadership and social capital facilitated collective actions, and directing response initiatives from other levels of the health system to the community. Support for community decision-making and action were identified as more important than funder-led short-term initiatives like building temporary treatment units, and this should be prioritized to mitigate health shocks in future.
